# Emergence of Vancomycin-Resistant *Enterococcus faecium* at an Australian Hospital: A Whole Genome Sequencing Analysis

**DOI:** 10.1038/s41598-018-24614-6

**Published:** 2018-04-19

**Authors:** Kelvin W. C. Leong, Louise A. Cooley, Tara L. Anderson, Sanjay S. Gautam, Belinda McEwan, Anne Wells, Fiona Wilson, Lucy Hughson, Ronan F. O’Toole

**Affiliations:** 1 School of Medicine, University of Tasmania, Hobart, Tasmania Australia; 2 Royal Hobart Hospital, Hobart, Tasmania Australia; 3 Tasmanian Infection Prevention and Control Unit, Department of Health and Human Services, Hobart, Tasmania Australia; 4 Trinity College Dublin, Department of Clinical Microbiology, Dublin, Ireland

## Abstract

In 2015, a marked increase in vancomycin-resistant *Enterococcus faecium* (VREfm) isolation was detected at the Royal Hobart Hospital, Australia. The primary objective of this work was to examine the dynamics of VREfm transmission using whole genome data mapped to public health surveillance information. Screening and clinical isolates of VREfm from patients were typed for the specific vancomycin-resistance locus present. Of total isolates collected from 2014–2016 (*n* = 222), 15.3% and 84.7% harboured either the *vanA* or the *vanB* vancomycin-resistance locus, respectively. Whole-genome sequencing of 80 isolates was performed in conjunction with single-nucleotide polymorphic (SNP) analysis and *in silico* multi-locus sequence typing (MLST). Among the isolates sequenced, 5 phylogenetic clades were identified. The largest *vanB* clade belonged to MLST sequence type ST796 and contained clinical isolates from VREfm infections that clustered closely with isolates from colonised patients. Correlation of VREfm genotypes with spatio-temporal patient movements detected potential points of transmission within the hospital. ST80 emerged as the major *vanA* sequence type for which the most likely index case of a patient cluster was ascertained from SNP analyses. This work has identified the dominant clones associated with increased VREfm prevalence in a healthcare setting, and their likely direction of transmission.

## Introduction

Healthcare-associated infections (HAIs) are a frequent adverse event which affect patients worldwide while receiving care. A systematic review conducted by the World Health Organization determined that the pooled prevalence of HAIs ranged from 5.7% to 19.1% in low- and middle-income countries^1^. A study by Graves and colleagues established that approximately 175,153 (95% CI: 155,911–195,168) cases of HAI occur among admissions to Australian hospitals annually causing an estimated 854,289 extra bed days (95% CI: 645,091–1,096,244) at a mean valuation of $1,005 per bed day^2^. The effect of HAIs includes prolonged hospital stay, recurrent illness, and a higher financial burden incurred by health systems.

The leading causes of HAIs globally are the so-called ESKAPE pathogens comprising *Enterococcus faecium*, *Staphylococcus aureus*, *Klebsiella pneumoniae*, *Acinetobacter baumannii*, *Pseudomonas aeruginosa*, and *Enterobacter* species for which antibiotic resistance is common^3^. A survey published by the Australian Group on Antimicrobial Resistance (AGAR) Australian Enterococcal Sepsis Outcome Program (AESOP), reported 1058 episodes of enterococcal bacteraemia in 2016, of which 56.2% and 39.0% were caused by *E. faecalis* and *E. faecium*, respectively^4^.

Due to the high frequency of penicillin resistance in *E. faecium* isolates (86.3–95.9% in Australian hospital cases)^5^, vancomycin is the primary antibiotic for the treatment of *E. faecium* infections. In 1994, the first isolate of vancomycin-resistant *E. faecium* (VREfm) in Australia was collected from a liver transplant recipient at a Melbourne hospital^6^ following its prior emergence in Europe and the USA in the 1980s^7,8^. Since then, the rate of vancomycin resistance in *E. faecium* in Australia has grown to become one of the highest in the world at 48.7–56.8% of clinical isolates in 2015^5^. The 30 day all-cause mortality rates in VREfm and vancomycin-susceptible *E. faecium* bacteraemia cases in Australia were 28.7% and 25.6%, respectively, in 2016^4^.

**Figure 1:**
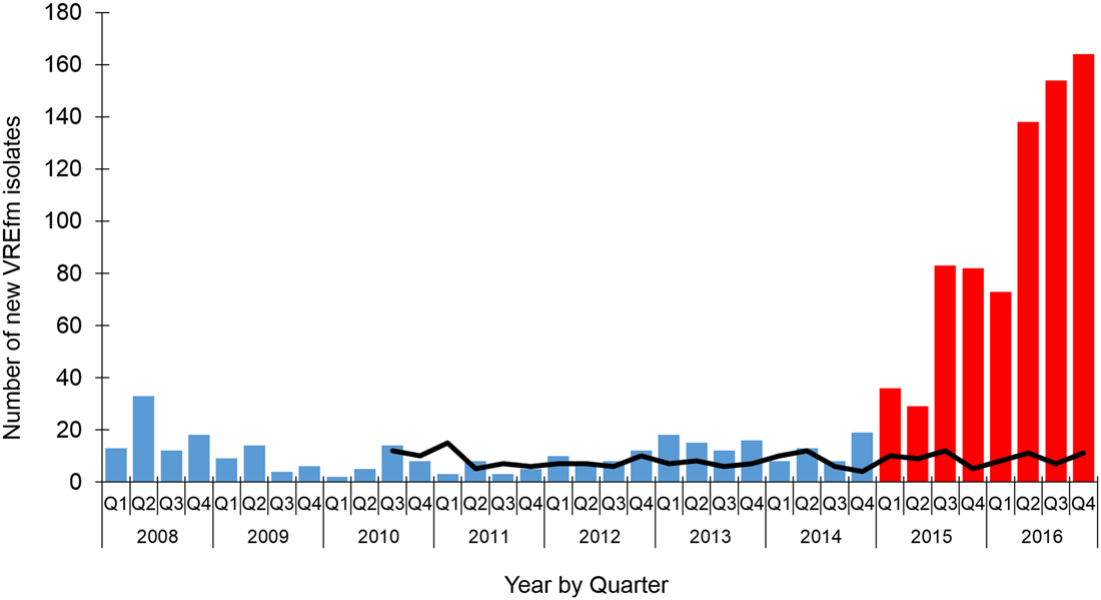
Number of newly identified vancomycin-resistant enterococcal (VRE) isolates (clinical and/or screening) in Tasmanian healthcare settings, 2008–2016. The number of new VRE isolates reported from Q1, 2008 to Q4, 2016 (columns). Since Q1, 2015, a marked increase in VRE isolates is indicated (red columns). The number of healthcare-associated *Staphylococcus aureus* bacteraemia cases (HA-SAB) is shown for comparison (black line).

In Tasmania, vancomycin-resistant enterococci (VRE) is a notifiable disease under the Public Health Act 1997^9^. A marked increase in VREfm notifications occurred from the beginning of 2015 (Fig. 1)^10^. In terms of infection control, hand hygiene compliance in Tasmania exceeded the national benchmark from 2013 onwards and continued to improve prior to, and during the increased notifications of VREfm^11^. Notably, there has been no corresponding increase in healthcare-associated bacteraemias in Tasmania due to *Staphylococcus aureus* (Fig. 1) which has previously been used as a sentinel marker for hand hygiene compliance^12^. In relation to antimicrobial stewardship, vancomycin has seen decreasing usage rates in Australian hospitals from 2010–2015, and no increase in usage in Tasmanian hospitals during this period^13^. Therefore, a greater understanding of the epidemiology of VREfm in Tasmania is required to support future initiatives to reduce the VREfm healthcare burden in the state.

**Figure 2:**
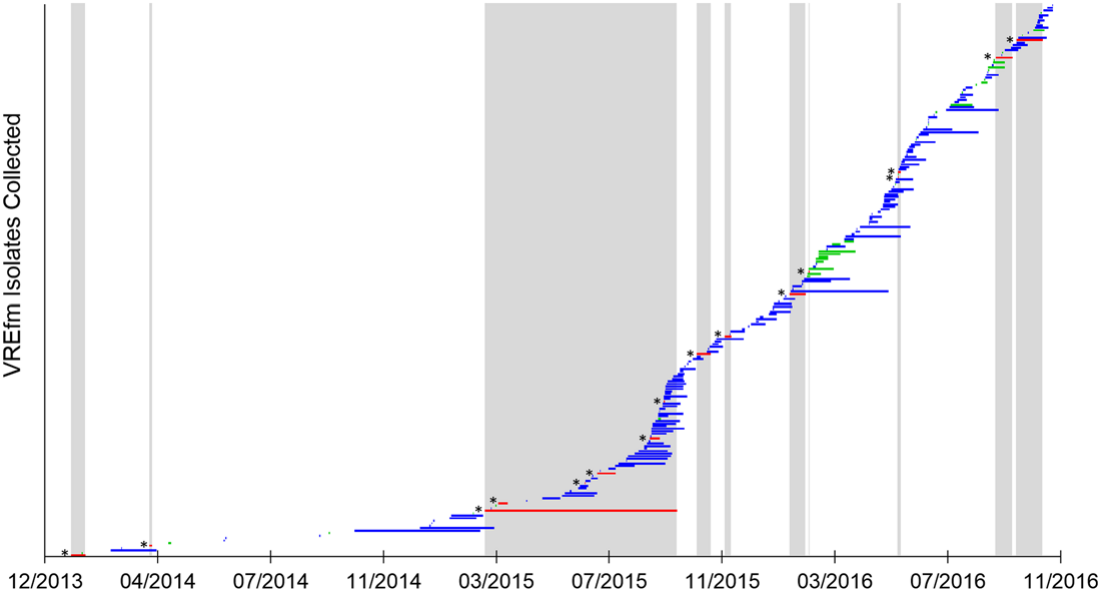
Temporal distribution of vancomycin-resistant *Enterococcus faecium* (VREfm) isolates (*n* = 222) collected from patients at the Royal Hobart Hospital from 2014–2016. Each horizontal bar represents the period of time between patient hospital admission and VREfm sample collection. The selection criteria of VREfm isolates for whole-genome sequencing were based on the temporal overlap (vertical silver columns) of clinical isolates (red bars with*) (*n* = 16) with *vanB* screening VREfm isolates (blue bars). Also included in this study were VREfm isolates that tested positive for the *vanA* locus (green bars) (*n* = 34).

In this work, we performed next-generation sequencing of VREfm isolates collected at the Royal Hobart Hospital (RHH), which is Tasmania’s major tertiary referral centre that manages over 400 beds. We then correlated genomic information with epidemiological data to better define the hospital spread of VREfm.

## Results

### Patient isolate and epidemiological data collection

Based on application of the VRE screening criteria of the Multi-Resistant Organism Screening and Clearance Protocol for the Tasmanian Health Service (described in Methods section), 222 VREfm isolates were collected from colonised and/or infected inpatients at the RHH from 2014–2016. 15.3% and 84.7% of total isolates collected harboured either the *vanA* or the *vanB* vancomycin-resistance locus, respectively. From this, a total of 80 unique patient VREfm isolates were processed for whole-genome sequencing. These included 14 retrievable clinical isolates from 2014 (*n* = 2), 2015 (*n* = 7) and 2016 (*n* = 5). All screening isolates from 2014 (*n* = 4) and 2016 (*n* = 55) that met the selection criteria were included for sequencing. In addition, 7 out of 57 *vanB* screening isolates that were temporally related to 2015 clinical cases were sequenced.

### *In silico* multi-locus sequence typing (MLST) and vancomycin-resistance locus detection

The MLST of each of the 80 sequenced VREfm isolates was assessed *in silico* from the genome assemblies using the Multi-Locus Sequence Typing (MLST) tool from the Center for Genomic Epidemiology (CGE) database (http://www.genomicepidemiology.org/). The isolates belonged to sequence types ST796 (*n* = 47), ST80 (*n* = 16), ST1421 (*n* = 10), ST203 (*n* = 4), ST78 (*n* = 1), ST192 (*n* = 1), and ST555 (*n* = 1) (Table 1). The clinical isolates were predominantly ST796 (*n* = 12), with the remaining clinical isolates belonging to sequence types ST78 (*n* = 1) and ST192 (*n* = 1).

**Table 1:** Distribution of MLST sequence types and vancomycin-resistance loci identified for the *Enterococcus faecium* (VREfm) isolates from the Royal Hobart Hospital that were whole-genome sequenced (*n* = 80).

MLST	*van* gene	2014	2015	2016	Total
ST80	A	1	1	13	15
ST80	A/B			1	1
ST1421	A	1		9	10
ST203	A		2	2	4
ST555	A	1			1
ST796	B	3	10	34	47
ST78	B		1		1
ST192	B			1	1
		6	14	60	80

Using the ResFinder tool from the CGE database (http://www.genomicepidemiology.org/), the type of vancomycin-resistance locus present was confirmed from the genome assemblies of the isolates. 98.8% of the 80 sequenced isolates harboured either the *vanA* (*n* = 26) or *vanB* (*n* = 53) locus, exclusively (Table 1). One isolate, 16S_RHH028 harboured both the *vanA* and *vanB* loci. The *vanA* isolates belonged to four MLST sequence types (ST80, ST203, ST555, and ST1421). The *vanB* isolates belonged to ST796, ST192, and ST78 and included all of the clinical isolates sequenced (*n = *14) (Table 1).

### Phylogenetic analysis

**Figure 3:**
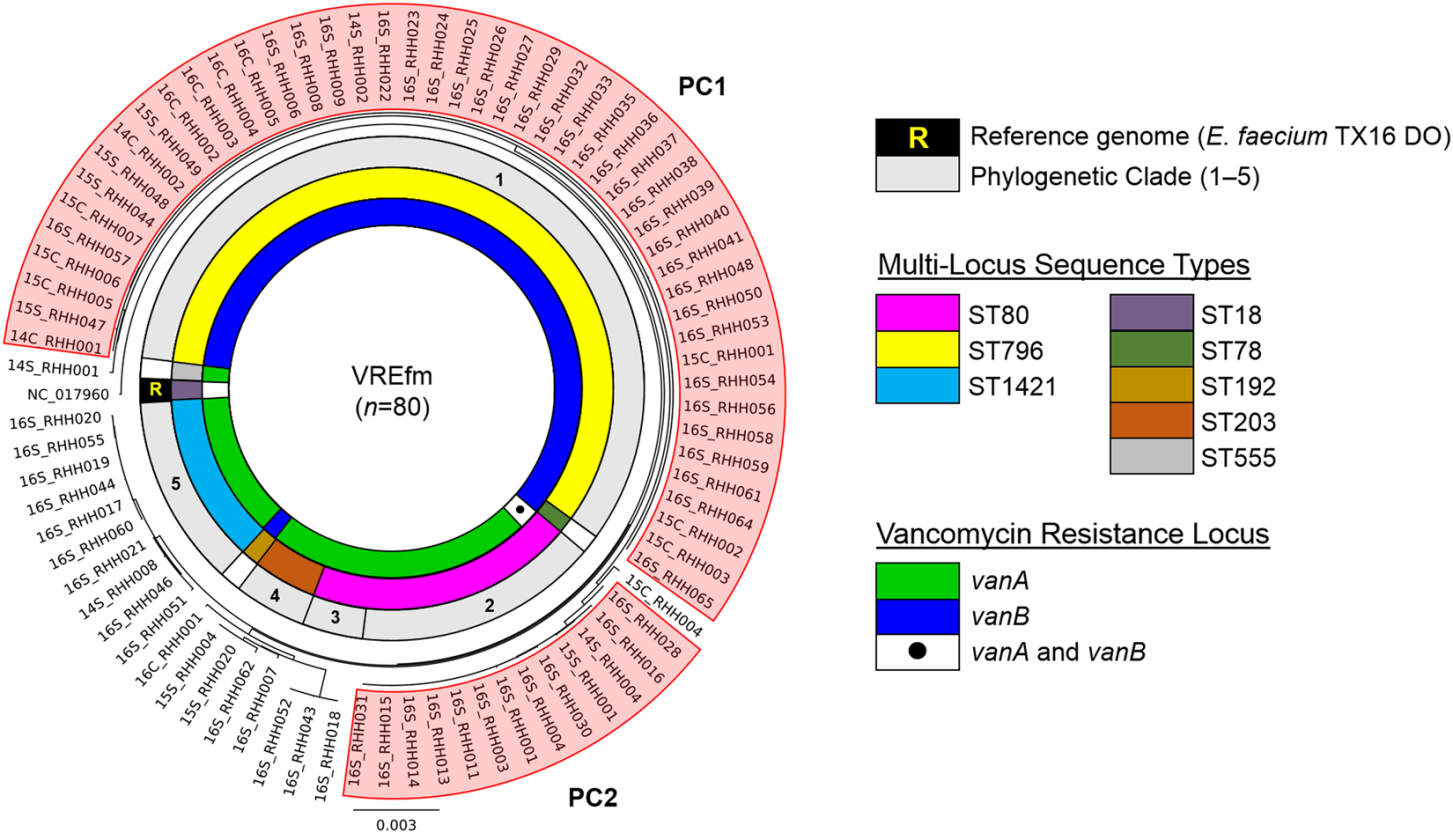
Phylogenetic analysis of vancomycin-resistant *Enterococcus faecium* (VREfm) isolates collected from patients at the Royal Hobart Hospital from 2014–2016 that underwent whole-genome sequencing (*n* = 80). A SNP-based maximum-likelihood (PhyML) phylogenetic tree was generated using *E. faecium* DO TX16 as the reference genome (NC017960) to root the tree. Branches of the phylogenetic tree revealed five major phylogenetic clades (PC). The whole genome data enabled *in silico* determination of multi-locus sequence types (MLST) and vancomycin resistance loci.

The genome sequence of the *E. faecium* ST18 DO (TX16) strain, which comprises 2,698,137 base-pairs, was used as the primary reference for the mapping of the raw FASTQ sequence reads of the VREfm isolates. An average read depth of 84.2-fold (minimum mean read depth of 28.6-fold) was obtained providing a mean coverage of 84.2% with respect to the *E. faecium* DO (TX16) reference genome (Supplementary Table S1). A maximum-likelihood phylogenetic tree, rooted to the *E. faecium* DO (TX16) reference genome, was constructed using PhyML (Fig. 3). The phylogenetic tree of the VREfm isolates was resolved into five major clades based on patristic distances.

The largest clade, Phylogenetic Clade 1 (PC1), comprised 47 *vanB*-harbouring isolates which belonged to ST796 (Fig. 3). The ST80 isolates (*n* = 16) were spread across two separate clades: Phylogenetic Clade 2 (PC2) (*n* = 13); and Phylogenetic Clade 3 (*n* = 3). One of the isolates belonging to PC2 harboured both the *vanA* and *vanB* vancomycin-resistance loci (Fig. 3). Phylogenetic Clade 4 consisted of isolates which belonged to ST203 (*n* = 4). The 10 isolates in Phylogenetic Clade 5, which lack the *pstS* gene, were assigned to a recently-designated MLST sequence type, ST1421, by the PubMLST database (https://pubmlst.org/efaecium/).

### Phylogenetic Clade 1 (PC1)

**Figure 4:**
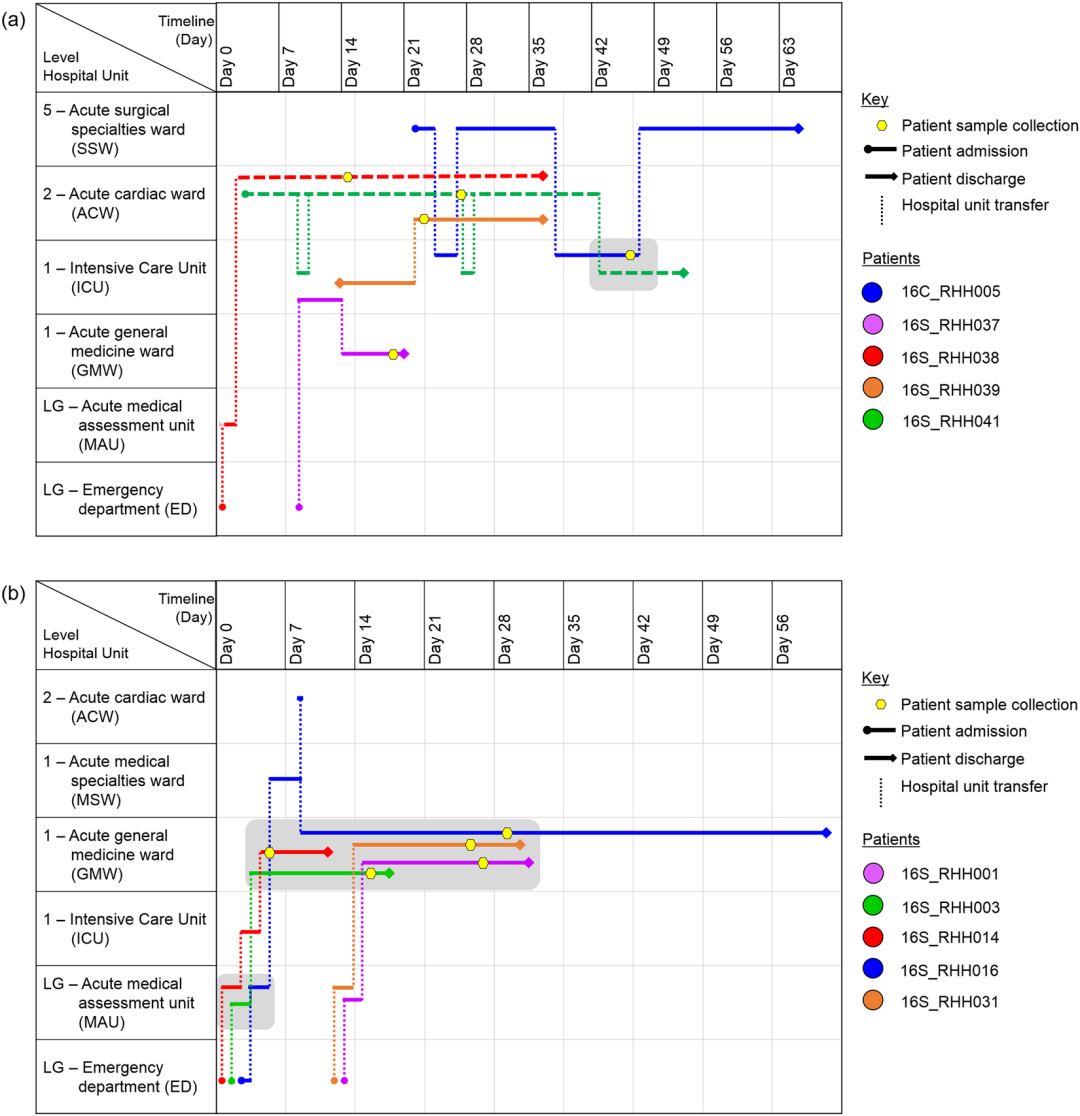
Spatio-temporal location of patients who tested positive for vancomycin-resistant *Enterococcus faecium* (VREfm) at the Royal Hobart Hospital. (**a**) Transmission of *vanB* ST796 VREfm from closely-related screening isolates belonging to Phylogenetic Clade 1 (PC1) to clinical patient 16C_RHH005. (**b**) Transmission of *vanA* ST80 VREfm among patients with isolates belonging to Phylogenetic Clade 2 (PC2). The movement of patients following admission to the Royal Hobart Hospital through to date of discharge are indicated with respect to time (x-axis) and hospital location (y-axis). The colour of each line represents each patient (dotted line – screening patient; solid line – clinical patient).

PC1 includes both clinical isolates (*n* = 12) and closely-related screening isolates (*n* = 35). These isolates all belong to sequence type ST796 and harbour the *vanB*-resistance locus (Fig. 3). Analysis of the clinical isolates from 2016 *i.e*. 16C_RHH002, 16C_RHH003, 16C_RHH004, and 16C_RHH005, did not reveal any points of intersection in terms of shared time and location in the hospital between the respective patients (Supplementary Table S1). Therefore, the field of investigation was expanded to include screening isolates. Clinical isolate from patient 16C_RHH005 was found to overlap with a number of screening isolates in terms of patient movements within the hospital. A closely-related screening isolate was collected from patient 16S_RHH038 who was admitted to the Emergency Department (ED) on day 0, moved on the same day to the acute medical admissions unit (MAU), before being transferred on day 2 to the acute cardiac ward (ACW) (Fig. 4). During this patient’s stay in the ACW, another patient 16S_RHH041 was admitted to the same ward on day 3. In the ACW, both patient 16S_RHH038 and patient 16S_RHH041, were identified with VREfm on day 14 and day 27, respectively. Patient 16S_RHH038 was discharged from the hospital on day 36, while patient 16S_RHH041 remained in the ACW until day 42 before being transferred to the Intensive Care Unit (ICU). On day 22, clinical patient 16C_RHH005 was admitted and transferred to the acute surgical specialties ward (SSW). The patient was moved to the ICU on days 23–26 and again on days 37–47. During the second visit to the ICU, patient 16C_RHH005 tested positive for VREfm on day 46. This suggests VREfm transmission may have occurred in the ICU in the direction from patient 16S_RHH041 to patient 16C_RHH005, the latter of whom presented with clinical symptoms of a urinary tract infection.

**Figure 5:**
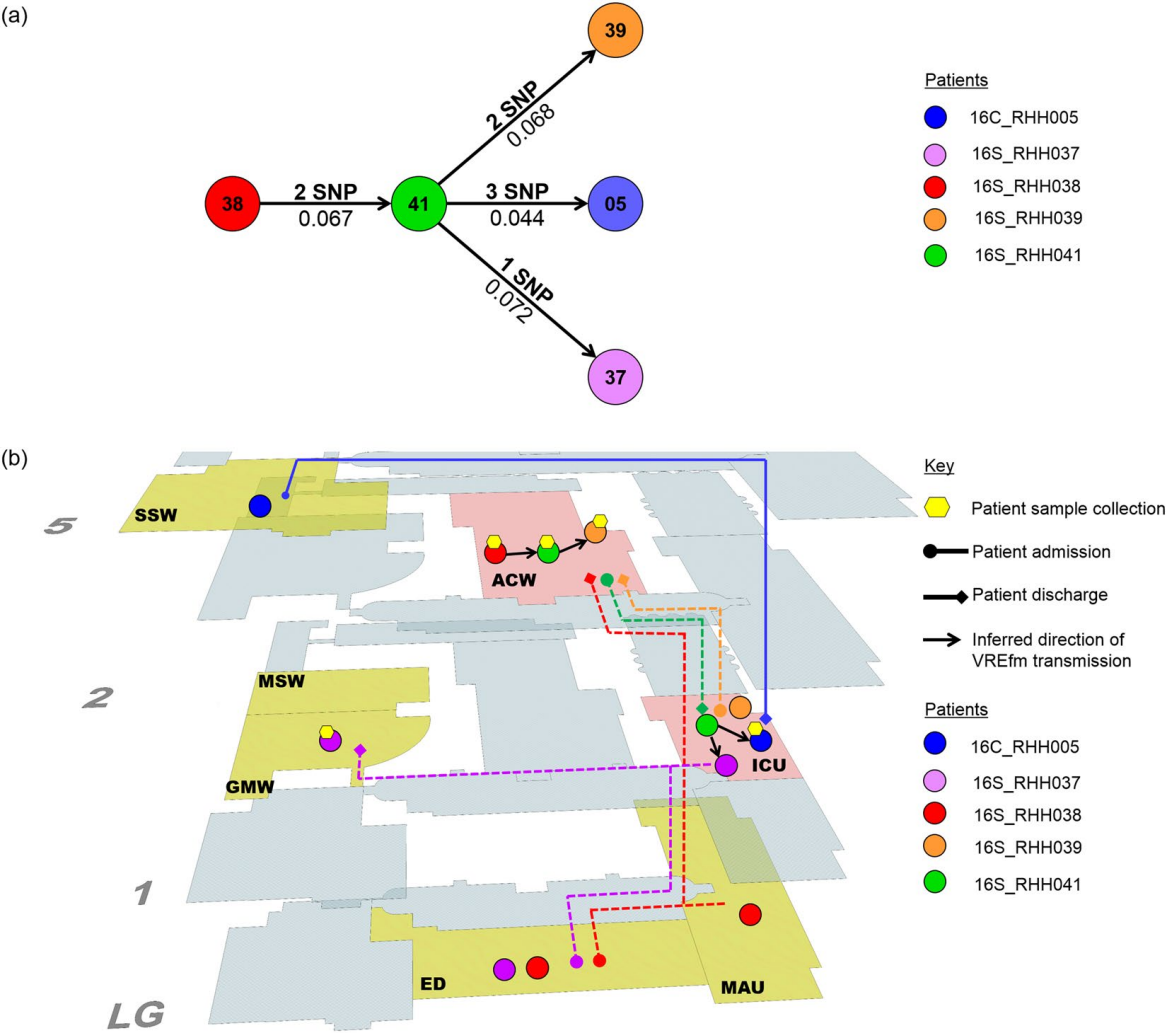
Vancomycin-resistant *Enterococcus faecium* (VREfm) transmission analysis based on single nucleotide polymorphism (SNP) genotypes of Phylogenetic Clade 1 (PC1). (**a**) Inferred VREfm transmission directions are represented by line connections between patients. Phylogenetic relationship is measured by differences in unique SNPs (numbers above connecting lines) and patristic distances between the isolates (numbers below connecting lines). (**b**) Correlation of SNP genotype data with epidemiological data revealed likely direction of transmission and potential points of transmission within the hospital. Potential points of transmission of PC1 *vanB* ST796 VREfm are indicated (pink areas). The Intensive Care Unit (ICU) was identified as the most likely location where transmission of VREfm occurred to patient 16C_RHH005 who presented with clinical symptoms of a urinary tract infection. Additional hospital units where patients from this cluster were located are indicated (yellow areas). Hospital ward abbreviations: ACW, acute cardiac ward; ED, emergency department; GMW, acute general medicine ward; ICU, intensive care unit; MAU, acute medical admissions unit; MSW, acute medical specialties ward; SSW, acute surgical specialties ward.

Based on the pairwise SNP analysis, VREfm transmission most likely occurred in the direction from patient 16S_RHH038 to patient 16S_RHH041 (2 SNP differences) (Fig. 5). We also determined that patient 16S_RHH041 was involved in VREfm transmission in the direction of patients 16S_RHH039 (2 SNP differences), 16S_RHH037 (1 SNP difference), and the patient who presented with clinical symptoms, patient 16C_RHH005 (3 SNP differences) (Fig. 5). From the available spatio-temporal data (Fig. 5), it is inferred that the ICU represented a potential location where transmission of VREfm to patient 16C_RHH005 could have occurred.

### Phylogenetic Clade 2 (PC2)

PC2 contains 12 screening isolates which were all sequence type ST80 and harboured the *vanA* locus, and a thirteenth ST80 isolate which harboured both the *vanA* and *vanB* vancomycin-resistance loci (Fig. 3). The first patient in the clade, patient 16S_RHH014, was admitted to the ED on day 0. On the same day, the patient was transferred to the MAU, and later to a single room in the ICU on day 2 (Fig. 4). Patient 16S_RHH014 was then moved to a shared room in the acute general medicine ward (GMW) for days 4–11. Upon transfer to the GMW, patient 16S_RHH014 was screened for VREfm colonisation on day 5 and when tested positive, was moved from a shared room to a single isolation room.

On day 1, patient 16S_RHH003 was admitted through the ED before being transferred to the MAU, and then to the GMW for days 3–17. On day 15, patient 16S_RHH003 was screened and found to be colonised with VREfm in this hospital unit (Fig. 4). Patient 16S_RHH016 was admitted to the ED on day 3, transferred to the MAU on day 4, before moving to the acute medical specialties ward (MSW) for days 5–8. On day 8, the patient visited the ACW before transferring to the GMW until being discharged on day 61. Patient 16S_RHH016 was found to be VREfm positive on day 29. Based on the epidemiological data, patients 16S_RHH014 and 16S_RHH003 shared time in the MAU for 1 day and also in the GMW for 7 days (Fig. 4). While in the GMW, both patients were co-located with patient 16S_RHH016 on days 8–12. As the patients were colonised with sequence type ST80 *vanA* VREfm isolates, this was suggestive of possible VREfm transmission involving these patients.

**Figure 6:**
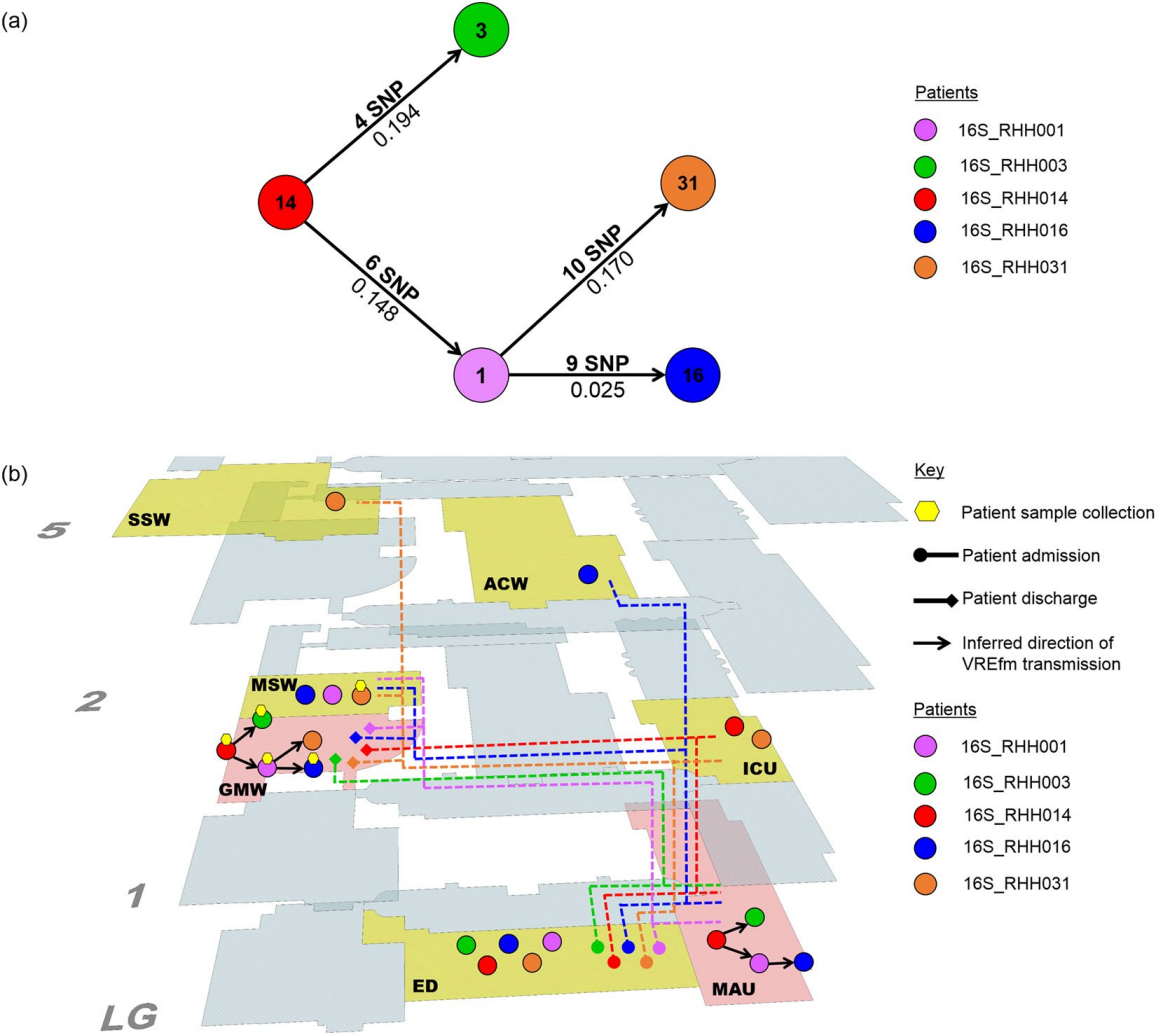
Vancomycin-resistant *Enterococcus faecium* (VREfm) transmission analysis based on single nucleotide polymorphism (SNP) genotypes of Phylogenetic Clade 2 (PC2). (**a**) Inferred VREfm transmission directions are represented by line connections between patients. Phylogenetic relationship is measured by differences in unique SNPs (numbers above connecting lines) and patristic distances between the isolates (numbers below connecting lines). (**b**) Correlation of SNP genotype data with epidemiological data revealed likely direction of transmission and potential points of transmission within the hospital. Potential points of transmission of PC2 *vanA* ST80 VREfm are indicated (pink areas). The acute medical admissions unit (MAU) and the acute general medicine ward (GMW) represented potential points where transmission of VREfm may have occurred from the index patient 16S_RHH014 in the direction of patient 16S_RHH003. Additional hospital units where patients from this cluster were located are indicated (yellow areas). Hospital ward abbreviations: ACW, acute cardiac ward; ED, emergency department; GMW, acute general medicine ward; ICU, intensive care unit; MAU, acute medical admissions unit; MSW, acute medical specialties ward; SSW, acute surgical specialties ward.

Based on the pairwise SNP data, patient 16S_RHH014 was involved in the transmission of VREfm to two other patients, 16S_RHH003 (4 SNP differences) and 16S_RHH001 (6 SNP differences) (Fig. 6). Further possible transmission occurred in the direction from patient 16S_RHH001 to patients 16S_RHH016 (9 SNP differences) and 16S_RHH031 (10 SNP differences) (Fig. 6). Combining the pairwise SNP data with the available spatio-temporal data, it is inferred that the acute medical admissions unit (MAU) represented the first potential point of VREfm transmission between patients 16S_RHH014 and 16S_RHH003. The acute general medicine ward (GMW) represented the second potential point where transmission of VREfm may have occurred from patient 16S_RHH014 in the direction of patients 16S_RHH003 and 16S_RHH001, and subsequently in the direction of patients 16S_RHH016 and 16S_RHH031 (Fig. 6).

## Discussion

In this study, we investigated the epidemiology of VREfm in Tasmania’s tertiary referral hospital following an increase in detection of VREfm isolates from hospital inpatients. The study illustrates changing epidemiology from sporadic, generally genetically diverse isolates in 2014, to the emergence of endemic VREfm with two large phylogenetic clades.

The study period also coincides with an increasing prevalence of *vanA* VREfm in Australia. In 2011, among vancomycin non-susceptible *E. faecium* bloodstream isolates (MIC > 4 mg/L), the prevalence of the *vanA* resistance locus was only 1.9% (2/107), compared to 97.2% (104/107) for *vanB*^14^. However in 2016, the prevalence of the *vanA* locus in vancomycin non-susceptible *E. faecium* isolates had increased to 43.0% (83/193)^4^. These data indicate a shift towards a higher prevalence of the *vanA* locus among *E. faecium* isolates from enterococcal bacteraemia episodes in Australia. Here, we found that of the total vancomycin-resistant *E. faecium* isolates collected at the Royal Hobart Hospital (RHH) from 2014 to 2016 (*n* = 222), 15.3% harboured the *vanA* resistance locus with the remainder harbouring the *vanB* resistance locus. None of the *E. faecium* isolates from Tasmania in the 2011 AGAR study contained the *vanA* locus, although it is important to note that the number of Tasmanian isolates included was low (*n* = 4)^14^.

Whole genome sequencing was performed on 80 of the VREfm isolates from the RHH from 2014–2016, which included all of the *vanA* isolates during this period. *In silico* determination of MLST revealed that the *vanA* isolates belonged to four MLST sequence types (ST80, ST203, ST555 and ST1421) (Table 1). While detected at low levels in 2014 and 2015, ST80 was the dominant sequence type among the RHH *vanA* isolates in 2016 (Table 1). The recently emerged prominence of ST80 in Tasmania is consistent with the national trend where there has been an increase in the number of *vanA* VREfm isolates that belong to this sequence type from 2.4% (5/253) in 2014 to 12.8% (51/400) in 2016^4,15^.

The dominant MLST sequence type among the *E. faecium vanB* locus containing isolates was ST796 (96.0%). This sequence type was discovered in Australia in 2012 and identified as the cause of a large clonal outbreak of VREfm colonisation in a Melbourne neonatal intensive care unit in 2013^16^. By 2015, ST796 had largely replaced ST203 as the dominant *vanB* sequence type among patient episodes of *E. faecium* bacteraemia in Melbourne hospitals^17^. Across Australia, ST796 was responsible for 14.0% of all sequence typed *E. faecium* bacteraemias in 2016, the second highest among MLST typeable isolates^4^. In this study, ST796 was identified in 12 out of 14 clinical isolates of VREfm. These included the three clinical isolates from patients 16C_RHH003, 16C_RHH004, 16C_RHH005 which belonged to Phylogenetic Clade 1 (PC1) but did not overlap with one another with respect to patient location and period of time (Supplementary Table S1). Incorporation into our analysis of screening isolates from patients who were co-incident in the hospital with the clinical isolate patients enabled us to detect a likely transmission network involving patient 16C_RHH005 and four VREfm colonised patients (Fig. 5).

Based on our whole genome variant and patristic distance analyses, transmission occurred involving the first member of the network, colonised patient 16S_RHH038, and patient 16S_RHH041 (Fig. 5). This was then followed by transmission in the direction from 16S_RHH041 to patients 16S_RHH039, 16S_RHH037, and to patient 16C_RHH005 who presented with urinary tract symptoms (Fig. 5). From the available spatio-temporal data, the intensive care unit (ICU) represented the most likely location where transmission of VREfm in the direction from patient 16S_RHH041 to patient 16C_RHH005 occurred (Fig. 5). Intensive care facilities have previously been identified as locations within hospitals that are associated with an increased risk of transmission of HAI pathogens to patients^18,19^. In a meta-analysis of ICU studies, the pooled cumulative incidence and density of HAIs were 34.7 per 100 patients (95% CI: 23.6–47.7) and 47.9 per 1000 patient days (95% CI: 36.7–59.1), respectively^20^.

In terms of the *vanA* locus containing VREfm isolates, a phylogenetic clade, PC2, which contained 12 ST80 isolates was identified from the sequence data. Again, epidemiological and whole genome variant analysis revealed a potential transmission network in which screening patient 16S_RHH014 was the most probable index case and was involved in VREfm transmission in the direction of patients 16S_RHH003 and 16S_RHH001 (Fig. 6). The latter patient was subsequently involved in secondary transmission in the direction of patients 16S_RHH031 and 16S_RHH016 (Fig. 6). This cluster occurred despite the index patient, 16S_RHH014, being managed under transmission-based precautions. Patient 16S_RHH014 was co-located with patient 16S_RHH003 in two of the hospital units, MAU for 1 day and the GMW for 7 days (Fig. 4), and therefore, transmission of VREfm to patient 16S_RHH003 may have occurred at either of these locations. The main role of the Acute Medical Admissions Unit (MAU) at the Royal Hobart Hospital is to assess acute patients who have been admitted through the Emergency Department. In a previous study by Kumar and colleagues at the Royal Liverpool and Broadgreen University Hospitals, they determined that the majority of *Clostridium difficile* transmission events, that occurred between wards, originated from the hospital’s Acute Medical Assessment Unit (AMAU)^21^. They cited high patient turnover rates and high patient contact rates as factors involved in the spread of *C. difficile* at the medical assessment unit.

Although all of the *vanA* VREfm isolates, and most of the *vanB* VREfm isolates, examined in this study were collected from asymptomatic patients, it is important to note that VREfm colonisation generally precedes infection. It is estimated that there may be ten times as many colonised as infected patients^22^. Patient colonisation has the propensity to establish VREfm reservoirs and enable further silent dissemination of VREfm to high-risk patients, such as in ICUs, directly, or indirectly through healthcare workers, or via environmental contamination. This finding is supported by previous studies that reported the role of patient colonisation as a source of VREfm transmission and the importance of ongoing active surveillance screening as an effective infection control strategy to prevent onward transmission^16^. Our study demonstrates the utility of examining isolates from asymptomatic colonised patients to link epidemiological gaps between patients from whom clinical isolates have been obtained. Incorporation of screening isolates that were co-incident in both time and place allowed improvement in our understanding of VREfm transmission within a healthcare institution.

In summary, since 2014, the VREfm epidemiological profile has changed at the Royal Hobart Hospital from sporadic detection to endemic. ST796 and ST80 have emerged as the major sequence types, a profile consistent with other states in Australia. Whole genome sequencing provided the ability to delineate closely-related isolates of VREfm, belonging to the same MLST sequence type. Furthermore, in tandem with epidemiological information, SNP-based variant analysis enabled the tracing of VREfm transmission networks in a hospital setting.

## Methods

### VREfm isolate collection

In Tasmania, VRE is a notifiable disease under the Public Health Act 1997^9^. The identification of new isolates of VRE from screening or clinical specimens is publicly reported through the Tasmania Infection Prevention and Control (TIPCU) Healthcare Associated Infection Surveillance Program. VRE infection is defined as a positive culture for VRE obtained from either a sterile site or from a non-sterile site where VRE specific antibiotic therapy was administered by a clinician^23^. VRE colonisation is defined as a positive culture for VRE associated with a non-sterile site isolate where VRE specific antibiotic therapy was not administered by a clinician^23^. A state-wide VRE screening and clearance protocol has been in place in Tasmania since 2010 with management of patients identified to be colonised or infected with VRE under transmission-based precautions whilst inpatients. Based on the Multi-Resistant Organism Screening and Clearance Protocol for the Tasmanian Health Service, the following patients are screened for VRE in Tasmania: direct transfers from any intrastate, interstate or overseas acute or long term healthcare facility; patients with an overnight admission in the previous 3 months to any intrastate acute or long term healthcare facility; patients with an overnight admission in the previous 12 months to any interstate or overseas acute or long term healthcare facility; patients with a ‘History-VRE’ alert; patients with a self-reported or healthcare facility reported history of VRE; patients identified to be a VRE contact^24^.

During 2014–2016, there were 222 patients identified to have VREfm colonisation or infection at the Royal Hobart Hospital (RHH) which consisted of 16 clinical and 206 screening isolates (one per patient). Stored isolates were retrieved and cultured on blood agar plates. Identification was confirmed using Bruker Biotyper (MALDI-TOF MS) (Bruker Daltonic GmbH, Leipzig, Germany). Antibiotic susceptibility testing of the VREfm screening and clinical isolates was performed using agar disc diffusion assay and Etest strips (bioMérieux). The zone diameters of inhibition and the minimum inhibitory concentrations were interpreted using the published EUCAST clinical breakpoints for Enterococcus species (http://www.eucast.org/clinical_breakpoints/)^25^. The Cepheid Xpert^®^ *vanA/vanB* assay was conducted on the isolates to detect vancomycin resistance locus. The criteria used for the selection of isolates for genome sequencing were as follows: all clinical isolates; *vanB* screening isolates that temporally overlapped with clinical isolates from date of patient hospital admission through to date of collection of VREfm positive sample; all *vanA* isolates (Fig. 2). This produced 80 isolates which consisted of 14 clinical isolates which were retrievable and 66 screening isolates for genome sequencing. Ethics approval for this study was obtained from the Tasmanian Health and Medical Human Research Ethics Committee (Reference number: H0016214). Isolates for this observational non-interventional study were obtained from routine testing performed according to clinical requirements. No research participants were specifically recruited for this study. All experiments were performed in accordance with relevant guidelines and regulations.

### Genomic DNA extraction and purification

Enterococcal isolates from patient samples were cultured in 15 mL thioglycollate broth (TM0935) (Thermo Fisher Scientific, Waltham, Massachusetts, USA) at the RHH and were analysed at the School of Medicine, University of Tasmania (UTAS). Cell pellets were harvested by centrifugation at 10,000× g for 8 minutes. The pellets were initially resuspended in a mixture of 30 µL lysozyme (50 mg/mL) (Muramidase, VWR Chemicals, Radnor, PA, USA) and 600 µL phosphate buffered saline (PBS) and the suspension was incubated at 37 °C for 1 hour. Genomic DNA was extracted from 200 µL of lysate according to the DNeasy Blood and Tissue Kit protocol (Qiagen, Hilden, Germany). The eluted DNA obtained was further treated with 2 µL of RNase (100 mg/mL) (Qiagen, Hilden, Germany) and incubated at room temperature for 1 hour. 100 µL of the eluted DNA solution was further purified with the High Pure PCR Template Preparation Kit (Roche, Basel, Switzerland) according to the manufacturer’s protocol to obtain purified DNA in a volume of 50 µL. For each sample, DNA was quantified using the Qubit dsDNA (double-stranded DNA) HS (high sensitivity) Quantification Kit and a Qubit 2.0 Fluorometer (Life Technologies, Carlsbad, CA, USA), and normalised to a concentration of 0.2 ng/µL with purified water.

### DNA library preparation and whole-genome sequencing

Nextera XT DNA libraries were prepared with half reaction volumes using the Illumina Nextera XT DNA Library Preparation Kit (Illumina Inc., San Diego, CA, USA) according to the manufacturer’s protocol. 2.5 µL of input DNA (0.2 ng/µL) were tagmented using the Applied Biosystems Veriti 96-Well thermal cycler (Thermo Fisher Scientific, Foster City, CA, USA), and neutralised with 2.5 µL of neutralisation tagmentation buffer (NT buffer). Illumina sequencing adaptors and index primers were added to the tagmented DNA via PCR amplification to generate a dual-indexed library for each sample. The DNA amplicon library was purified with Agencourt AMPure XP beads (Beckman Coulter, Brea, CA, USA) to remove impurities such as free index primers, short DNA fragments, and enzymes. The cleaned DNA libraries were quantified using Qubit dsDNA HS Quantification kit and a Qubit 2.0 Fluorometer (Life Technologies) and normalised to 10 µL to form a pooled amplified library (PAL). The concentration of the PAL was determined by amplification of a 1:1000 dilution of the library by qPCR (quantitative PCR) using the Corbett Rotor-Gene 6000 real-time thermocycler (Qiagen, Hilden, Germany). PCR assays were prepared with the KAPA Library Quantification Kit (KAPA Biosystems Inc., Wilmington, MA, USA) following the manufacturer’s recommended protocol. Sequencing of the cleaned pool was carried out on a MiSeq sequencing instrument (Illumina, San Diego, CA, USA) with the MiSeq v2 Reagent Kit (Illumina) to generate 2 × 150-bp paired-end reads.

### Genome assembly and phylogenetic analysis

Raw sequence reads from whole-genome sequencing (WGS) were mapped to the annotated and publicly-available complete reference genome sequence of the *E. faecium* ST18 DO (TX16, NC017960) strain that was isolated in Texas, USA in 1998^26^ using Snippy (https://github.com/tseemann/snippy). Contiguous consensus sequences (contigs) produced from reference mapping were used for the detection of single nucleotide polymorphisms (SNPs) relative to the reference genome. The presence of a SNP was defined using a minimum nucleotide variant frequency of 90% being covered by at least 25 reads. To infer phylogeny, Snippy produced a FASTA alignment file of the core genome SNPs. Gubbins (https://github.com/sanger-pathogens/Gubbins) was used with the FASTA alignment file as input for the prediction and extraction of high SNP density which were representative of regions of recombination. The software was executed using the default options: the reference genome *E. faecium* DO strain was used in the alignment to root the phylogenetic tree; for phylogeny inference with maximum likelihood estimation, RaxML, was used; and phylogenetic tree convergence was set to a maximum of 5 iterations. A range of sliding window sizes between 100 and 10,000 bases were used to parse the genome sequence for the detection of recombination regions consisting of at least 3 SNPs. Sequences that exhibited less than 80% coverage, with respect to the reference genome, were excluded. A set of genome alignments with the recombination regions removed was then generated. The core genome alignment was used to re-construct a maximum-likelihood phylogenetic tree with the PhyML plugin in Geneious (Biomatters Ltd.)^26^, using a general time-reversible model with gamma correction (GTR-gamma).

### *De novo* genome assembly

Using the Velvet plugin in Geneious, the raw sequence read-pairs were assembled *de novo* with the Velvet optimiser to automatically determine the k-mer length in the range of 27 to 35 to optimise genome assembly. The genome assemblies were checked using Geneious to identify the presence of the seven Multi-Locus Sequence Typing (MLST) housekeeping genes. The assembled genome sequences were queried against the Multi-Locus Sequence Typing tool (http://www.mlst.net/databases) from the Center for Genomic Epidemiology (CGE) database (http://www.genomicepidemiology.org/) to determine the sequence type (ST) of the isolates. Genome sequences where the *pstS* gene was absent were submitted to the PubMLST database (https://pubmlst.org/efaecium/) for the designation of an MLST sequence type. Using the ResFinder antibiotic resistance gene analysis tool (https://cge.cbs.dtu.dk/services/ResFinder/) at the CGE database, another query was conducted to identify the presence of vancomycin resistance loci for each of the isolates.

### VREfm transmission analysis

For the application of VREfm genomic information to transmission analysis, epidemiological information was collected from the hospital’s electronic medical record and infection control database which included patient admission and discharge dates, specimen type and collection date, and patient ward/hospital movements during hospitalisation. The nomenclature used here for the de-identified patients is as follows: year of collection, specimen type (clinical or screening), hospital, and specimen number. For example, isolate 16S_RHH001 was collected in 2016 from a screening patient admitted to the RHH with a specimen number of 001.

For isolates belonging to a given phylogenetic clade, transmission analysis was commenced from the patient with the earliest date of hospital admission which was designated day 0. Subsequent events that occurred with respect to the phylogenetic clade are referred chronologically in relation to day 0. The movements within the hospital of patients whose isolates belonged to a given clade, and their VREfm sample collection dates, were then plotted against time. From this spatio-temporal analysis, it was possible to identify patients who were potentially epidemiologically linked through shared time and space in the hospital.

To determine direction of VREfm transmission, earlier typing techniques such as MLST did not provide a sufficient level of differentiation between isolates due to the limited number of gene sequences characterised. Therefore, a genome-wide SNP based pairwise analysis was used to infer recent transmission. For each cluster, the raw FASTQ sequences of the isolates were mapped against the consensus genome sequence of the index isolate. Pairwise SNP differences were calculated to establish the number of unique SNPs between the isolates after recombination regions had been removed from the genomes’ core region. For patients with a spatio-temporal overlap during their stay in the hospital, VRE transmission was inferred between isolates with the least number of unique SNP differences, proceeding in the direction of increasing SNP differences. A similar approach has been used previously with respect to *Mycobacterium tuberculosis*^27^ and *Clostridium difficile*^28^ and is based upon the premise that a low number of SNP differences between isolates is indicative of recent transmission.

### Data availability statement

The FASTQ sequence reads of the VREfm clinical isolates have been submitted to the European Nucleotide Archive (study accession number: PRJEB25449).

## Electronic supplementary material

Supplementary Table S1
