# Supplementary material for: Emergence of Vancomycin-Resistant Enterococcus faecium at an Australian Hospital: A Whole Genome Sequencing Analysis
Source: Sci Rep. 2018 Apr 19;8:6274. doi: 10.1038/s41598-018-24614-6 (PMC5908837; doi:10.1038/s41598-018-24614-6)
Supplement: Supplementary file 1 — Supplementary Table S1 [file 41598_2018_24614_MOESM1_ESM.pdf]

Emergence of Vancomycin-Resistant *Enterococcus faecium* at an Australian Hospital: A Whole Genome Sequencing Analysis

Kelvin W.C. Leong[1], Louise A. Cooley[1,2], Tara L. Anderson[2,3], Sanjay S. Gautam[1], Belinda McEwan[2], Anne Wells[3], Fiona Wilson[3], Lucy Hughson[3], and Ronan F. O'Toole[1,4,*]

[1]School of Medicine, University of Tasmania, Hobart, Tasmania, Australia

[2]Royal Hobart Hospital, Hobart, Tasmania, Australia

[3]Tasmanian Infection Prevention and Control Unit, Department of Health and Human Services, Hobart, Tasmania, Australia

[4]Trinity College Dublin, Department of Clinical Microbiology, Dublin, Ireland

[*]ronan.otoole@utas.edu.au

Supplementary Table S1. Sequence statistics and de-identified epidemiological information of the whole-genome sequenced vancomycin-resistant *Enterococcus faecium* (VREfm) isolates (*n*=80).

Isolate Name	MLST	Sample Type	Date of Admission	Date of Collection	Vancomycin Resistance Locus	% Coverage	No. of Aligned Bases	Mean Read Depth
14C_RHH001	ST796	Clinical - other sterile site	30/12/2013	14/01/2014	B	89.46	2413816	91.2
14C_RHH002	ST796	Clinical - urine	23/03/2014	26/03/2014	B	89.42	2412682	81.5
14S_RHH001	ST555	Screening	10/01/2014	11/01/2014	A	89.12	2404519	65
14S_RHH002	ST796	Screening	10/02/2014	31/03/2014	B	88.44	2386120	83.1
14S_RHH004	ST80	Screening	12/04/2014	15/04/2014	A	89.50	2414873	71.8
14S_RHH008	ST1421	Screening	30/09/2014	1/10/2014	A	89.05	2402743	80
15C_RHH001	ST796	Clinical - blood culture	29/06/2015	30/06/2015	B	89.35	2410856	79.2
15C_RHH002	ST796	Clinical - tissue	15/03/2015	5/10/2015	B	89.26	2408227	73.4
15C_RHH003	ST796	Clinical - urine	12/07/2015	1/08/2015	B	89.32	2410014	77.8
15C_RHH004	ST78	Clinical - urine	6/09/2015	17/09/2015	B	88.06	2375897	77.6
15C_RHH005	ST796	Clinical - urine	21/09/2015	22/09/2015	B	89.48	2414188	83.5
15C_RHH006	ST796	Clinical - urine	26/10/2015	10/11/2015	B	89.44	2413213	79.8
15C_RHH007	ST796	Clinical - urine	25/11/2015	2/12/2015	B	89.47	2413915	86.2
15S_RHH001	ST80	Screening	2/03/2015	3/03/2015	A	89.14	2405144	69.2
15S_RHH004	ST203	Screening	26/03/2015	27/03/2015	A	88.61	2390846	86.0
15S_RHH020	ST203	Screening	15/09/2015	18/09/2015	A	88.84	2397152	95.5
15S_RHH044	ST796	Screening	26/10/2015	30/10/2015	B	89.17	2406063	115.2
15S_RHH047	ST796	Screening	6/11/2015	18/11/2015	B	88.70	2393371	77.2
15S_RHH048	ST796	Screening	16/11/2015	15/12/2015	B	88.84	2397159	84.8
15S_RHH049	ST796	Screening	1/12/2015	16/12/2015	B	89.23	2407448	120.9
16C_RHH001	ST192	Clinical - tissue	2/02/2016	19/02/2016	B	90.93	2453291	76.8
16C_RHH002	ST796	Clinical - urine	22/02/2016	23/02/2016	B	89.24	2407799	85.8
16C_RHH003	ST796	Clinical - urine	27/03/2016	28/03/2016	B	89.49	2414509	87
16C_RHH004	ST796	Clinical - urine	26/04/2016	30/04/2016	B	89.36	2411088	92.4
16C_RHH005	ST796	Clinical - wound site	4/06/2016	26/06/2016	B	89.43	2412909	85.5
16S_RHH001	ST80	Screening	1/03/2016	19/03/2016	A	90.83	2450848	69.5
16S_RHH003	ST80	Screening	21/02/2016	6/03/2016	A	85.09	2295945	33.2
16S_RHH004	ST80	Screening	18/03/2016	27/03/2016	A	89.85	2424361	55
16S_RHH006	ST796	Screening	28/12/2015	31/12/2015	B	88.76	2394892	35.7
16S_RHH007	ST203	Screening	17/08/2016	19/02/2016	A	90.08	2430361	57.8
16S_RHH008	ST796	Screening	15/02/2016	17/03/2016	B	89.34	2410382	273.6
16S_RHH009	ST796	Screening	23/12/2015	7/01/2016	B	87.41	2358333	62.7
16S_RHH011	ST80	Screening	4/03/2016	14/03/2016	A	90.20	2433836	57.8
16S_RHH013	ST80	Screening	1/03/2016	9/03/2016	A	90.98	2454685	197.4
16S_RHH014	ST80	Screening	20/02/2016	24/02/2016	A	91.01	2455486	47.7
16S_RHH015	ST80	Screening	29/07/2016	8/08/2016	A	90.42	2439577	105.1
16S_RHH016	ST80	Screening	22/02/2016	20/03/2016	A	91.10	2457884	123.6
16S_RHH017	ST1421	Screening	24/05/2016	25/05/2016	A	91.45	2467385	63

16S_RHH018	ST80	Screening	27/05/2016	28/05/2016	A	88.57	2389615	110.9
16S_RHH019	ST1421	Screening	16/06/2016	16/06/2016	A	91.84	2477881	45.7
16S_RHH020	ST1421	Screening	6/07/2016	8/07/2016	A	91.76	2475899	57.3
16S_RHH021	ST1421	Screening	26/01/2016	8/02/2016	A	91.93	2480406	48
16S_RHH022	ST1421	Screening	4/08/2016	4/08/2016	A	89.19	2406352	66.6
16S_RHH023	ST796	Screening	5/02/2016	6/02/2016	B	88.78	2395298	53.4
16S_RHH024	ST796	Screening	12/01/2016	3/02/2016	B	89.45	2413453	54.8
16S_RHH025	ST796	Screening	15/01/2016	5/02/2016	B	89.35	2410744	73.5
16S_RHH026	ST796	Screening	5/02/2016	15/02/2016	B	89.34	2410480	83
16S_RHH027	ST796	Screening	15/02/2016	16/02/2016	B	89.19	2406430	72.2
16S_RHH028	ST80	Screening	4/03/2016	27/03/2016	A/B	89.27	2408495	86.2
16S_RHH029	ST796	Screening	17/02/2016	6/04/2016	B	88.02	2374907	96.7
16S_RHH030	ST80	Screening	31/03/2016	10/04/2016	A	89.99	2428043	81.6
16S_RHH031	ST80	Screening	4/03/2016	18/03/2016	A	90.96	2454356	168
16S_RHH032	ST796	Screening	12/04/2016	17/04/2016	B	88.22	2380431	90.9
16S_RHH033	ST796	Screening	3/02/2016	17/05/2016	B	89.45	2413391	80.4
16S_RHH035	ST796	Screening	24/05/2016	29/05/2016	B	89.40	2412072	178.4
16S_RHH036	ST796	Screening	1/04/2016	30/05/2016	B	88.47	2386996	107.7
16S_RHH037	ST796	Screening	28/05/2016	2/06/2016	B	88.95	2399974	134.9
16S_RHH038	ST796	Screening	17/05/2016	29/05/2016	B	89.14	2405092	90.4
16S_RHH039	ST796	Screening	29/05/2016	5/06/2016	B	88.72	2393680	139.5
16S_RHH040	ST796	Screening	20/05/2016	13/06/2016	B	88.52	2388378	100.3
16S_RHH041	ST796	Screening	19/05/2016	9/06/2016	B	88.80	2395828	87.4
16S_RHH043	ST80	Screening	28/06/2016	29/06/2016	A	85.50	2306773	28.6
16S_RHH044	ST1421	Screening	18/08/2016	19/08/2016	A	89.65	2418956	52.8
16S_RHH046	ST1421	Screening	6/09/2016	7/09/2016	A	90.88	2451958	49.6
16S_RHH048	ST796	Screening	29/08/2016	11/09/2016	B	91.72	2474697	93.6
16S_RHH050	ST796	Screening	14/09/2016	15/09/2016	B	88.10	2377078	70.3
16S_RHH051	ST1421	Screening	15/09/2016	16/09/2016	A	88.70	2393154	52.4
16S_RHH052	ST80	Screening	31/08/2016	18/09/2016	A	84.16	2270800	44.5
16S_RHH053	ST796	Screening	18/09/2016	2/10/2016	B	88.47	2387020	74.2
16S_RHH054	ST796	Screening	24/09/2016	5/10/2016	B	88.65	2392023	92.3
16S_RHH055	ST1421	Screening	6/10/2016	7/10/2016	A	89.37	2411387	47.2
16S_RHH056	ST796	Screening	30/09/2016	9/10/2016	B	91.27	2462683	83.9
16S_RHH057	ST796	Screening	12/10/2016	13/10/2016	B	88.93	2399416	72.6
16S_RHH058	ST796	Screening	22/10/2016	25/10/2016	B	89.47	2413996	90.7
16S_RHH059	ST796	Screening	24/10/2016	28/10/2016	B	89.15	2405337	83.8
16S_RHH060	ST1421	Screening	26/10/2016	27/10/2016	A	89.16	2405748	46
16S_RHH061	ST796	Screening	23/10/2016	30/10/2016	B	88.18	2379102	116.2
16S_RHH062	ST203	Screening	18/10/2016	30/10/2016	A	89.04	2402435	85.8
16S_RHH064	ST796	Screening	20/10/2016	3/11/2016	B	88.92	2399293	83.7
16S_RHH065	ST796	Screening	24/10/2016	3/11/2016	B	88.87	2397728	71.0
